# Management of Acute Diverticulitis and Incidental Abdominal Aortic Aneurysm in a 67-Year-Old Male: A Case Report of Balancing Priorities in a High-Risk Patient

**DOI:** 10.7759/cureus.78987

**Published:** 2025-02-14

**Authors:** Jesse O'Rorke, Greyson Butler, John A Moss

**Affiliations:** 1 Osteopathic Medicine, Lee Health, Fort Myers, USA; 2 Osteopathic Medicine, Lake Erie College of Osteopathic Medicine, Bradenton, USA; 3 Osteopathic Medicine, Lake Erie College of Osteopathic Medicine, Lakewood Ranch, USA; 4 Vascular Surgery, Lee Health, Fort Myers, USA

**Keywords:** hinchey classification, management of abdominal aortic aneurysms, screening of aaa, st-segment elevation myocardial infarction (stemi), type ii endoleak

## Abstract

Diverticular disease and abdominal aortic aneurysms (AAAs) represent distinct but significant clinical entities often associated with advanced age. Diverticulitis, a common complication of diverticular disease, can result in perforation and systemic complications, while AAAs, frequently asymptomatic, carry substantial morbidity and mortality risks if undetected or untreated. Advances in imaging have improved the early identification of these conditions, yet the simultaneous management of both presents unique challenges requiring multidisciplinary coordination.

A 67-year-old male with a history of ST-elevation myocardial infarction, hypertension, hyperlipidemia, and smoking presented with left lower quadrant abdominal pain and abnormal outpatient computed tomography (CT) findings. Imaging revealed Hinchey 1a diverticulitis with a microperforation and an incidental 6.5 cm saccular AAA. The patient received conservative treatment for diverticulitis with intravenous antibiotics and transitioned to oral antibiotics upon clinical improvement. Following resolution, the patient underwent successful endovascular aneurysm repair (EVAR) for the AAA, which was complicated by a type II endoleak identified postoperatively. Blood pressure management and hydration addressed acute kidney injury, and the patient recovered well with a multidisciplinary follow-up planned.

This case underscores the importance of imaging in diagnosing coexisting conditions, particularly in high-risk populations. Management required balancing the risks of treating acute diverticulitis with the need for prompt intervention for a large, saccular AAA. The conservative approach to diverticulitis, followed by elective EVAR, reflects a patient-centered strategy consistent with current guidelines. Furthermore, it underscores the critical role of adhering to screening recommendations for high-risk populations, as timely detection of asymptomatic conditions like AAAs can prevent life-threatening complications.

## Introduction

Diverticular disease of the colon is a common gastrointestinal condition in Western populations, with prevalence rates ranging from 5% to 45%, and its incidence increases with advanced age and obesity [1-3]. While many patients remain asymptomatic, a subset will develop acute diverticulitis, which can lead to complications such as perforation, abscess formation, and fistula formation [4]. Several classification systems are used to stratify diverticulitis cases, helping clinicians assess disease severity and guide treatment decisions. These include the Hinchey, Modified Hinchey, American Association for the Surgery of Trauma (AAST), and World Society of Emergency Surgery (WSES) classifications [5, 6]. Specifically, the Hinchey system includes four stages: Stage I involves localized pericolic abscesses (Ia, confined inflammation; Ib, localized abscess); Stage II features distant, walled-off abscesses (pelvic, intraabdominal, or retroperitoneal); Stage III denotes purulent peritonitis due to perforation; and Stage IV involves fecal contamination and resultant peritonitis from free perforation [5, 7, 8]. In recent decades, improved imaging techniques have facilitated earlier recognition of complications and incidental findings, broadening the scope of care required for these patients. Additionally, these advances have provided deeper insights into the underlying pathophysiology of diverticular disease and underscore the importance of integrative management approaches.

Abdominal aortic aneurysms (AAAs) represent another significant health concern, often detected incidentally during imaging for unrelated conditions. In a retrospective cohort study it was found to be incidentally discovered in roughly 1% of the 89,000 abdominal CT, ultrasound, and MRI scans studied [9]. These aneurysms frequently remain asymptomatic until their expansion or rupture, events that carry a high risk of morbidity and mortality [10]. Risk factors for AAA development include male gender, advanced age, smoking history, hypertension, and a family history of aneurysms, all of which influence screening and management strategies [10, 11]. Guidelines for managing AAAs emphasize regular monitoring and surgical intervention when the risk of rupture outweighs procedural risks, particularly in aneurysms larger than 5.5 cm or those expanding >1 cm/year [12, 13]. The coincidental detection of a large AAA during the workup of a patient with acute diverticulitis presents a unique clinical challenge, necessitating a multidisciplinary approach to timing and strategy for both conditions. Moreover, optimizing patient outcomes requires careful consideration of comorbidities and individualized treatment planning, with close coordination among gastroenterology, vascular surgery, and other specialties.

## Case presentation

**Figure 1:**
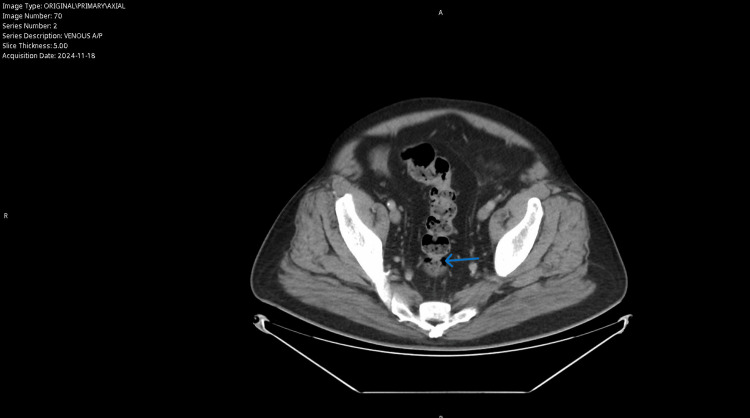
The patient’s abdominal CT scan, showing evidence of the micro-perforation of his sigmoid colon as designated by the arrow

**Figure 2:**
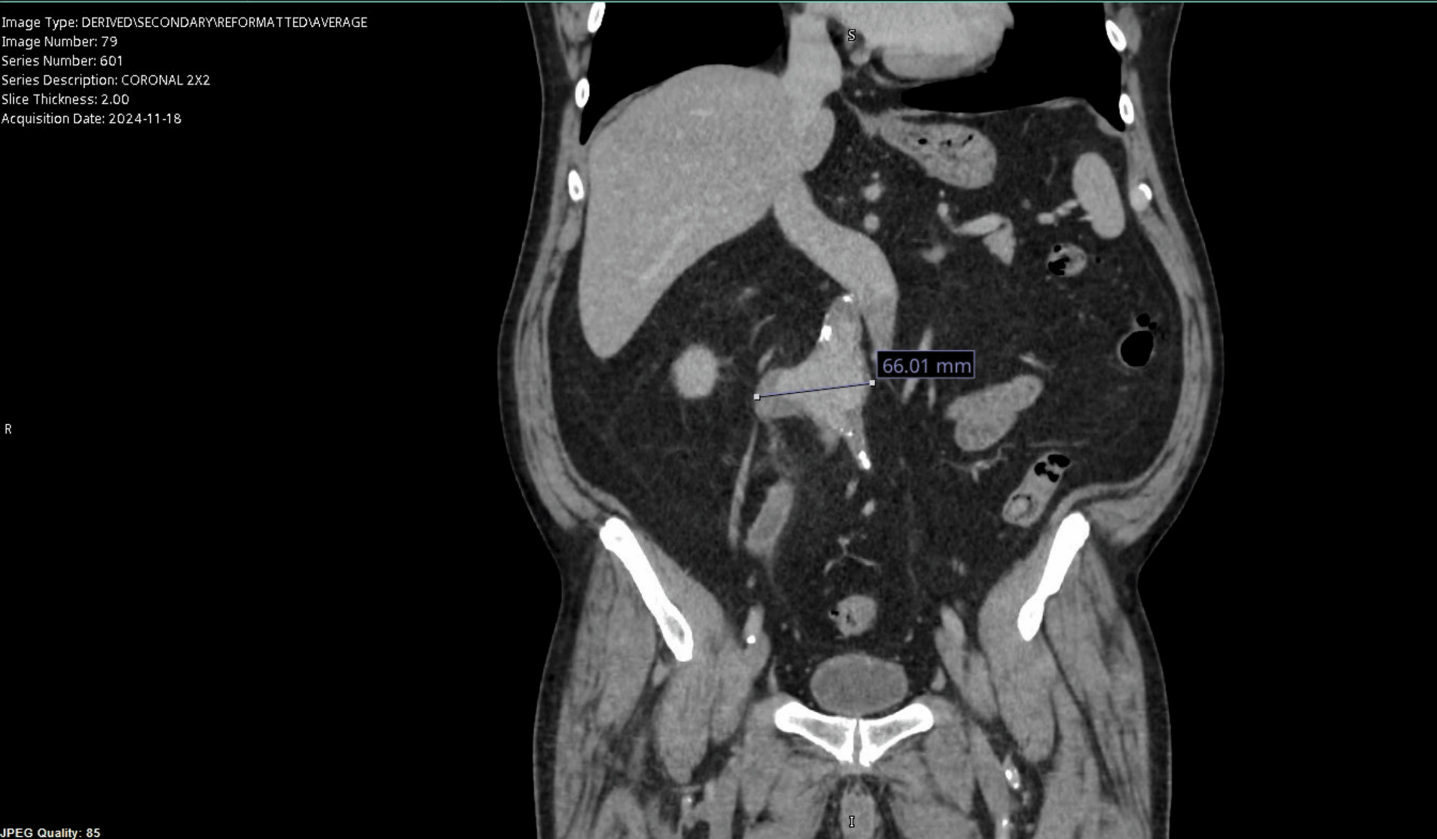
The patient’s abdominal CT scan, showing evidence of the abdominal aortic aneurysm with a saccular component, measuring >6 cm

A 67-year-old male with a past medical history of ST segment elevation myocardial infarction status post percutaneous coronary intervention (PCI) x2, hypertension, hyperlipidemia, and seizure disorder presented to the emergency department with the complaint of abnormal outpatient abdominal imaging. The patient went to his primary care group that morning and they advised him to go to the emergency department. He had an abdominal computed tomography (CT) scan that showed multiple abnormalities; the scan was ordered because of his complaint of left lower quadrant “on and off” abdominal pain that had been occurring for about a year; he reported that in the last three weeks, it had progressed. The CT scan showed likely diverticulitis of the sigmoid colon with a small perforation, without abscess formation, as demonstrated in Figure 1. The CT scan was also remarkable for a large distal abdominal aortic aneurysm (AAA) measuring 2.4 cm x 4.1 cm x 6.5 cm with a right-sided saccular component, as demonstrated in Figure 2. The aneurysm was approximately 5 cm below the renal arteries and without iliac artery involvement; there was no evidence of leak or rupture. The patient denies any previous history or knowledge of the aneurysm. He was a former smoker who smoked 1 pack per day of cigarettes. He quit at the time of his heart attack 20 years ago and now smokes a cigar daily.

At the time of presentation to the emergency department, he was bradycardic with a heart rate of 50 beats per minute and had evidence of an acute kidney injury with a creatinine of 1.48 mg/dL and a blood pressure of 163/72 mmHg. He had a white blood cell count that was within normal limits and denied fever, chills, chest pain, shortness of breath, and palpitations. The patient was started on Intravenous fluids and 3.375 grams of piperacillin-tazobactam every 6 hours for the perforated class Hinchey 1a diverticulitis. The vascular surgery team was consulted and their recommendation was to perform an endovascular repair of the AAA when the patient finishes his course of antibiotics. The cardiology team was consulted and cleared the patient for endovascular repair at moderate cardiac risk, with the approval to stop his clopidogrel, which was part of his dual antiplatelet therapy regimen, for one week prior to endovascular repair, if deemed necessary. On the following day, the general surgery team was consulted and they recommended that the patient be advanced to a regular diet, be transitioned to oral antibiotics, and have a colonoscopy 6-8 weeks following his discharge. They reported no surgical intervention was indicated at that time from their standpoint as it was the patient’s first episode of early uncomplicated diverticulitis. At the time of that consultation the following morning, the patient reported that his abdominal pain had resolved. The patient reported no new complaints on the second day of his admission and was transitioned to 875-125 milligrams of amoxicillin-clavulanate to be taken every 12 hours for 5 days. He was deemed stable for discharge at this time and left with his family. 

**Figure 3:**
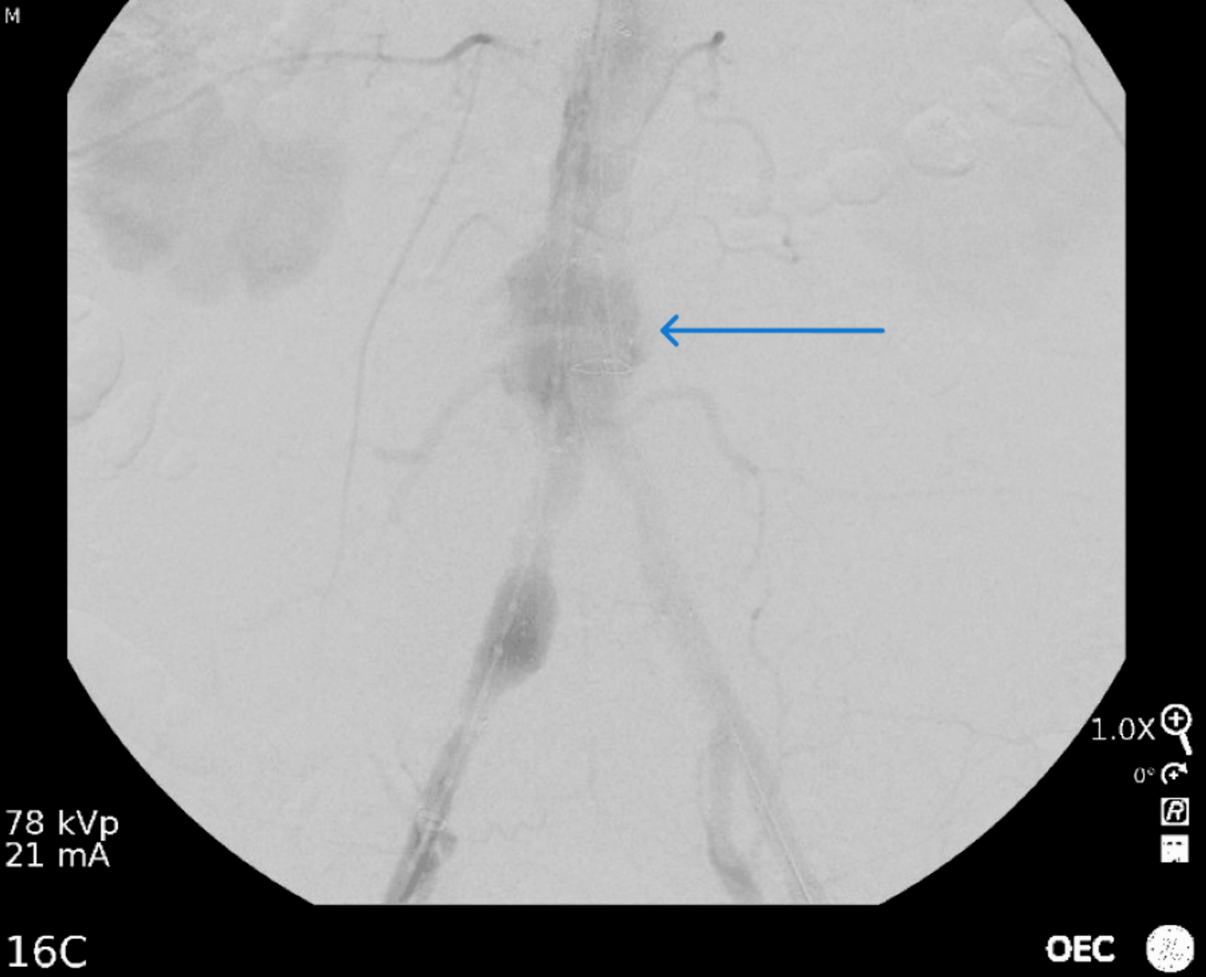
Perioperative imaging showing evidence of the type II endoleak from a lumbar branch, as indicated by the arrow

He was seen in the outpatient office of the vascular surgery team after he finished his antibiotic regimen and was scheduled for endovascular aneurysm repair (EVAR) 5 days after that visit. There were no physical examination changes at that time and the patient had no new subjective complaints. The patient presented to the hospital the following Monday and underwent EVAR with an excellent result. There was evidence of a delayed type-2 endoleak, which was identified postoperatively, but with no evidence of type I endoleak (Figure 3).

After the procedure, the patient became acutely hypertensive with a systolic blood pressure of up to 200 mmHg. He was started on a nicardipine drip and the blood pressure subsequently became well-controlled and back to the patient’s baseline of a systolic in the 140-mmHg range. Aggressive intravenous hydration was continued in an effort to resolve the patient's acute kidney injury. Overnight, the patient was stable and had no new complaints the following morning. The vascular surgery team, among other specialists, visited the patient in the ICU over the following 3 days. The patient was without any new subjective complaints, his groin access site had healed well, and he was determined stable for discharge on his third day after the EVAR. On discharge, he was sent home on 50 milligrams of hydralazine for blood pressure control along with his other blood pressure medications. He was advised to follow up with the vascular surgery team at their outpatient office within 1 week, the nephrologist in 2-3 weeks post-discharge to assess the status of his recovery from his newly discovered baseline stage IIIa chronic kidney disease (glomerular filtration rate (GFR) 41 on basic metabolic panel), and the gastroenterology team in 6-8 weeks for a follow-up colonoscopy following his acute diverticulitis episode.

## Discussion

The patient’s presentation of acute diverticulitis with a small perforation (Hinchey 1a) highlights a common yet potentially serious complication of diverticular disease. His clinical symptoms of left lower quadrant abdominal pain, combined with CT findings, confirmed the diagnosis and severity, demonstrating the utility of CT imaging in accurately assessing the extent of the disease [14]. The Hinchey classification system is a widely accepted method for categorizing diverticulitis severity and guiding treatment. This patient’s case, classified as Hinchey 1a, allowed for conservative management with intravenous piperacillin-tazobactam to target colonic flora, addressing gram-positive, gram-negative, and anaerobic pathogens [5, 8]. While antibiotic treatment has proven to be effective in cases of uncomplicated diverticulitis (Hinchey 1a), studies suggest that an observation-only approach is also successful in most cases [15-17]. Planning for a follow-up colonoscopy within 6-8 weeks was a key step to rule out underlying malignancy, as diverticulitis can mask or coexist with colorectal cancer in some cases [18]. This approach is particularly important given that this was the patient’s first episode of diverticulitis, and an underlying pathology should be excluded.

Abdominal aortic aneurysms (AAAs) are often asymptomatic until expansion or rupture, making their incidental discovery a pivotal opportunity for early intervention [10]. Current screening protocols recommend one-time abdominal ultrasonography for men aged 65 to 75 who have ever smoked, given the high prevalence of undiagnosed AAAs in this population [19]. This patient, a 67-year-old male with a history of smoking and hypertension, had several established risk factors for AAA development but had not yet undergone screening. Notably, his aneurysm was classified as saccular, a morphology associated with higher rupture risk compared to the more common fusiform type [20, 21]. This is because the localized outpouching of saccular AAAs creates areas of concentrated wall stress that weakens the vessel wall [22]. While the incidental discovery of his AAA through CT imaging was unplanned, it allowed for timely surgical planning and prevented progression to rupture, which carries an 85% mortality rate, of which 66% die before reaching the hospital [23]. This case highlights the importance of adhering to AAA screening protocols, especially in high-risk populations, to reduce the morbidity and mortality associated with undiagnosed aneurysms.

In addition to the saccular morphology, the patient’s AAA measured 6.5 cm in its largest dimension, exceeding the commonly accepted threshold of 5.5 cm for recommending surgical intervention in asymptomatic patients [12, 13]. These characteristics, combined with the patient’s comorbidities such as hypertension, made him a strong candidate for endovascular aneurysm repair (EVAR), a less invasive alternative to open surgical repair [24]. Importantly, the decision to delay EVAR until after the resolution of his acute diverticulitis reflected a careful balance of risks, as active inflammation or infection can significantly increase perioperative complications. This approach is consistent with clinical guidelines emphasizing the importance of addressing coexisting conditions prior to elective aneurysm repair to minimize adverse outcomes [25].

EVAR is a minimally invasive procedure used to treat AAAs by inserting a stent graft through the femoral arteries to reinforce the weakened aortic wall and exclude the aneurysm from circulation [24]. Unlike open surgical repair, which involves a large abdominal incision and direct repair of the affected aortic segment with a synthetic graft, EVAR avoids the need for open surgery by utilizing fluoroscopic guidance to deploy the stent graft within the aneurysm [24]. This approach is associated with reduced perioperative morbidity and mortality, shorter hospital stays, and faster recovery times compared to open repair, making it particularly advantageous for older patients or those with significant comorbidities [26, 27]. Additionally, EVAR is often the preferred option for patients with suitable aneurysm anatomy, such as sufficient infrarenal neck length, neck diameter, and infrarenal neck angulation [28]. However, EVAR is not without limitations, as long-term surveillance is required to monitor for complications such as endoleaks, graft migration, sac enlargement, or infection [29-31]. For this patient, the ch oice of EVAR over open repair was influenced by his advanced age, history of myocardial infarction, and chronic hypertension.

An endoleak is a complication following EVAR in which blood continues to flow into the aneurysm sac, preventing its complete exclusion from circulation and potentially leading to sac expansion or rupture. Endoleaks are classified into five types based on their source and mechanism. Type I endoleaks occur when there is inadequate sealing at the proximal or distal ends of the graft, allowing blood to flow into the aneurysm sac due to poor attachment. Type II endoleaks, the most common type, arise from retrograde flow into the aneurysm sac from collateral vessels, such as the lumbar or inferior mesenteric arteries, and are often managed conservatively unless sac expansion occurs. Type III endoleaks are caused by mechanical failure of the graft, such as fabric tears or disconnections between graft components, which require urgent repair. Type IV endoleaks result from the porosity of the graft material, allowing blood to seep through the graft wall, and are typically transient, resolving as the graft seals. Type V endoleaks, or "endotension," occur when the aneurysm sac expands without a visible source of blood flow and may require further intervention to prevent rupture (Table 1) [32, 33]. Identifying and managing endoleaks is critical to the success of EVAR, with routine imaging surveillance playing a key role in early detection and guiding appropriate treatment.

**Table 1:** Classification of Endoleaks

Type of Endoleak	Source and Mechanism of the leak
Type I	Inadequate sealing at the proximal or distal ends of the graft, allowing blood to flow into the aneurysm sac due to poor attachment.
Type II	Retrograde flow into the aneurysm sac from collateral vessels, such as the lumbar or inferior mesenteric arteries
Type III	Mechanical failure of the graft, such as fabric tears or disconnections between graft components
Type IV	Result from porosity of the graft material, allowing blood to seep through the graft wall, and are typically transient, resolving as the graft seals
Type V	Occur when the aneurysm sac expands without a visible source of blood flow

## Conclusions

This case highlights the critical role of incidental findings in improving patient outcomes, particularly in high-risk populations. The patient presented with acute diverticulitis complicated by a small perforation, managed conservatively with antibiotics, underscoring the importance of timely imaging and adherence to established management guidelines for Hinchey 1a diverticulitis. The discovery of a large saccular abdominal aortic aneurysm (AAA) during this workup was alarming yet potentially life-saving, as the discovered saccular morphology and aneurysm size significantly increased the risk of rupture. The interdisciplinary approach, incorporating gastroenterology, vascular surgery, cardiology, and nephrology, exemplifies the necessity of coordinated care in managing complex, concurrent conditions to achieve optimal outcomes.

The patient underwent successful endovascular aneurysm repair (EVAR), demonstrating the efficacy of this minimally invasive technique for treating AAAs in patients with significant comorbidities. Colonic diverticular disease shares many risk factors with AAA, and its incidence also increases with age. Screening of individuals at risk of AAA or accelerated AAA growth may identify those who can benefit from treatment to prevent or slow aneurysm progression, in turn reducing mortality and morbidity associated with AAA. This case underscores the importance of adhering to AAA screening protocols in at-risk populations, as early detection enables timely intervention. Moreover, it highlights the value of multidisciplinary collaboration and personalized treatment strategies in managing the dual challenges of acute diverticulitis and a high-risk aneurysm, emphasizing the need for vigilance and comprehensive follow-up care to mitigate long-term complications.
